# Development of a Brazilian anticholinergic activity drug scale

**DOI:** 10.31744/einstein_journal/2019AO4435

**Published:** 2019-03-18

**Authors:** Raiany Thaimeny Nery, Adriano Max Moreira Reis

**Affiliations:** 1 Hospital Risoleta Tolentino Neves, Belo Horizonte, MG, Brazil.; 2 Faculdade de Farmácia, Universidade Federal de Minas Gerais, Belo Horizonte, MG, Brazil.

**Keywords:** Cholinergic antagonists, Anticholinergic agents, Weights and measures, Drug therapy, Antagonistas colinérgicos, Agentes anticolinérgicos, Pesos e medidas, Tratamento farmacológico

## Abstract

**Objective:**

To develop a scale of anticholinergic activity drugs used in Brazil, to be applied in health care and pharmacoepidemiology research.

**Methods:**

We performed a literature review on PubMed/MEDLINE^®^ to identify previously published scales of anticholinergic drugs. This scale started with anticholinergic drugs, and those with known anticholinergic activity as per the 4^th^ level, chemical-therapeutic subgroup, of the Anatomical Therapeutic Chemical classification. We also included drugs with high anticholinergic activity, as described in a list of potentially inappropriate medications for use in older adults, according to the 2015 American Geriatrics Society Beers Criteria. Drugs listed in at least two anticholinergic scales were added. Then we verified which drugs in the previous steps were marketed in Brazil. We assigned a score of 1, 2 and 3, based on their anticholinergic action.

**Results:**

A total of 273 anticholinergic drugs were identified, of which 125 were included in the scale. We identified 45 (36.0%) drugs with a score of 3, 13 (10.4%) with a score of 2, and 67 (53.6%) with a score of 1. Drugs for the nervous and respiratory systems were the most frequent in the scale. Eight drugs were not present in previous scales.

**Conclusion:**

The methodology used for development of the Brazilian anticholinergic activity scale is simple, systematized, reproducible and easy to update. The scale allows evaluating the impact of anticholinergic burden on health outcomes, and can potentially contribute to pharmacoepidemiology research, leading to more accurate measurements of anticholinergic activity.

## RESUMO

**Objetivo:**

Desenvolver uma escala de atividade anticolinérgica abrangendo os medicamentos utilizados no Brasil, para aplicação no cuidado em saúde e em pesquisas farmacoepidemiológicas.

**Métodos:**

Realizou-se revisão da literatura no PubMed/MEDLINE^®^para identificação das escalas de mensuração da atividade anticolinérgica. Iniciou-se a escala com os fármacos anticolinérgicos e aqueles com atividade anticolinérgica conhecida, relacionados segundo o nível 4, subgrupo químico, na classificação da *Anatomical Therapeutic Chemical* . Incluíram-se os fármacos com atividade anticolinérgica alta, descritos na lista de medicamentos potencialmente inapropriados para idosos, segundo o 2015 *American Geriatrics Society Beers Criteria* . Adicionaram-se os medicamentos que constavam em, no mínimo, duas escalas anticolinérgicas. Em seguida, verificaram-se os medicamentos constantes nas etapas anteriores comercializados no Brasil. A magnitude da atividade anticolinérgica foi estabelecida em escores com os valores de 1, 2 e 3.

**Resultados:**

Foram identificados 273 medicamentos com atividade anticolinérgica, sendo 125 incluídos na escala. Destes, 45 (36,0%) receberam pontuação 3, 13 (10,4%) tiveram pontuação 2, e 67 (53,6%) pontuação 1. A maioria dos medicamentos da escala atuava nos sistemas nervoso e respiratório. Oito fármacos não constavam em escalas prévias.

**Conclusão:**

A metodologia de desenvolvimento da escala brasileira de atividade anticolinérgica é simples, sistematizada, reprodutível e de fácil atualização. A escala permite avaliar o impacto da carga anticolinérgica nos resultados em saúde e pode contribuir com as pesquisas farmacoepidemiológicas, propiciando mensurações mais exatas da atividade anticolinérgica.

## INTRODUCTION

Drugs with anticholinergic activity are used to treat a great part of older adults, psychiatric patients, and individuals with Parkinson’s disease, in addition to being used to treat several chronic and acute health conditions.^(^ ^1^ ^-^ ^3^ ^)^ These drugs have an intrinsic anticholinergic activity, directly related to their chemical nature, or not related to their major therapeutic indication.^(^ ^2^ ^,^ ^3^ ^)^ Drugs with anticholinergic activity may have central (dizziness, nervousness, delirium and hallucinations) and peripheral (xerostomia, constipation, blurred vision and urinary retention) adverse effects.^(^ ^4^ ^)^

The anticholinergic burden refers to the cumulative effect of one or more drugs with anticholinergic activity.^(^ ^2^ ^)^ Evidence suggests that a high anticholinergic burden is associated with greater morbidity and mortality, longer lengths of stay, higher institutionalization rates, as well as functional and cognitive decline.^(^ ^2^ ^)^ Therefore, these drugs should be cautiously prescribed to older adults, who are more vulnerable to anticholinergic effects due to the use of multiple drugs, higher blood-brain membrane permeability, and age-related pharmacokinetic changes.^(^ ^3^ ^)^

Scales have been developed to be used in clinical practice to measure the anticholinergic burden of drugs and, therefore, their potential to cause adverse effects.^(^ ^1^ ^)^ Carnahan et al.,^(^ ^5^ ^)^ were among the first authors to propose a scale to rate drugs based on their anticholinergic potential. These scales are based on information from expert consensus reports, anticholinergic activity data, or a combination of both. The objective measurement of the anticholinergic burden can be performed by determining the serum anticholinergic activity (SAA) of the drug and testing its muscarinic receptor affinity.^(^ ^1^ ^)^

Strategies to reduce the anticholinergic burden may produce considerable health benefits.^(^ ^6^ ^)^ Anticholinergic risk scales provide healthcare professionals with a practical tool to prevent anticholinergic adverse effects in older adults, and are important for the development of strategies to optimize drug safety.^(^ ^1^ ^,^ ^5^ ^)^

Pharmacoepidemiology research is required for a better understanding of the benefits and risks of drug therapies, particularly in the elderly population. The development of implicit tools, in turn, can help guide the process of prescribing and simplifying drug schedules.^(^ ^7^ ^)^ Measuring exposure to anticholinergics is a method used in clinical practice and healthcare research, Investigations help understand how the anticholinergic overload can impact health outcomes, however they require appropriate methodologies and accurate measurements of exposure.^(^ ^8^ ^)^ The anticholinergic activity scales currently available were developed and validated in the US, Europe and Australia.^(^ ^1^ ^,^ ^3^ ^,^ ^8^ ^)^ However they do not comprise all drugs with anticholinergic activity, and do not account for the drugs available in different countries.^(^ ^1^ ^)^

## OBJECTIVE

To develop an anticholinergic activity scale comprising the drugs used in Brazil, to be applied in health care and pharmacoepidemiology research.

## METHODS

### Identification of anticholinergic activity rating scales

A literature review was conducted on PubMed/MEDLINE^®^ comprising the period between January 2006 and July 2017, using the Medical Subject Headings (MeSH) terms “cholinergic antagonists”, “anticholinergic”, “anticholinergic agents” and keywords “nicotinic antagonists”, “muscarinic antagonists”, “atropinic”, “scale”, “load”, “burden”, “risk”, “exposure” and “medication”. The search strategy used boolean operators *AND* and *OR* . The search was limited to articles in English and had the purpose of identifying anticholinergic activity rating scales.

The articles were selected by title and abstract, by two investigators. The eligible studies were subjected to a complete text analysis. The inclusion criterion was studies that featured an instrument to rate the anticholinergic burden of drugs.

A total of 11 anticholinergic scales with activity grading were identified and selected for data extraction and development of our scale: Anticholinergic Drug Scale (ADS),^(^ ^5^ ^)^ Anticholinergic Burden Classification (ABC),^(^ ^9^ ^)^ Clinician-Rated Anticholinergic Score (CrAS^),(^ ^10^ ^)^ Anticholinergic Risk Scale (ARS),^(^ ^11^ ^)^ Serum Anticholinergic Activity (SAA),^(^ ^12^ ^)^ Anticholinergic Cognitive Burden Scale (ACB),^(^ ^13^ ^)^ Anticholinergic Activity Scale (AAS),^(^ ^14^ ^)^ Anticholinergic Load Scale (ACL),^(^ ^15^ ^)^ Anticholinergic Effect on Cognition (AEC),^(^ ^16^ ^)^ Muscarinic Acetylcholine Receptor ANTagonist Exposure (MARANTE)^(^ ^4^ ^)^ and Anticholinergic Impregnation Scale (AIS).^(^ ^3^ ^)^

Until July 2017, three systematic reviews had been published aiming to identify anticholinergic activity rating scales, but only one of them described the scales and the associations between calculated anticholinergic burdens and clinical outcomes.^(^ ^17^ ^)^ The other two reviews provided tables with the name of the drugs with anticholinergic activity listed in the scales.^(^ ^1^ ^,^ ^8^ ^)^ Some investigations use the name Duran Scale, or Duran List, to refer to the table of 100 drugs rated as high or low activity, contained in the ADS,^(^ ^5^ ^)^ ABC,^(^ ^9^ ^)^ SAA,^(^ ^12^ ^)^ ARS,^(^ ^11^ ^)^ CrAS,^(^ ^10^ ^)^ AAS^(^ ^14^ ^)^ and ACL scales,^(^ ^15^ ^)^ developed by the authors of the systematic review, and based on said scales. It was supplemented by a search on Martindale: the complete drug reference,^(^ ^18^ ^)^ to clarify any discrepancies between scale scores.^(^ ^1^ ^,^ ^17^ ^,^ ^19^ ^)^ A table of 195 drugs was developed based on a systematic review, which also covered the ADS,^(^ ^5^ ^)^ ABC,^(^ ^9^ ^)^ SAA,^(^ ^12^ ^)^ ARS,^(^ ^11^ ^)^ CrAS,^(^ ^10^ ^)^ AAS^(^ ^14^ ^)^ and ACL scales,^(^ ^15^ ^)^ however rating them into high, medium and low anticholinergic activity. This table points out the discrepant scores found in the different scales.^(^ ^8^ ^)^

A table of drugs with definite, probable and possible anticholinergic effects was published during the study period, but it presented no activity grading.^(^ ^20^ ^)^ The Summated Anticholinergic Medications Scale (SAMS) includes only the anticholinergic drugs with high anticholinergic activity, listed in the 2012 American Geriatrics Society Beers Criteria^(^ ^21^ ^)^ and previous studies, and the only difference is that it states the minimum effective daily dose to calculate the anticholinergic burden.^(^ ^22^ ^)^ The Drug Burden index is a composite index measuring the anticholinergic and sedative burden considering the daily dose used, but with no specific list of anticholinergic drugs or activity grading.^(^ ^23^ ^)^

### Development of the anticholinergic activity rating scale

The anticholinergic activity rating scale was developed using the following steps:

1. Preparation of a preliminary list containing the anticholinergic drugs previously listed in the 4^th^ level of the Anatomical Therapeutic Chemical (ATC) system. These chemical groups were described by Puustinen et al.,^(^ ^24^ ^)^ and Brown et al.,^(^ ^25^ ^)^ and comprise anticholinergic drugs of frequent use in clinical practice. We also included therapeutic groups comprising drugs with known anticholinergic activity.^(^ ^2^ ^,^ ^24^ ^)^ The codes of the chemical groups corresponding to the drugs included in this step can be found in table 1 .
  **Table 1:** Anatomical Therapeutic Chemical (ATC) 4th level codes of the drugs included in the first step of development of the Brazilian anticholinergic activity scale

ATC code for anticholinergic drugs
A03AA − synthetic anticholinergics, esters with tertiary amino group
A03AB − synthetic anticholinergics, quaternary ammonium compounds
A03BA − belladonna alkaloids, tertiary amines
A03BB − belladonna alkaloids, semisynthetic, quaternary ammonium compounds
A03CA − synthetic anticholinergic agents in combination with psycholeptics
A03CB − belladonna and derivatives in combination with psycholeptics
A03DA − synthetic anticholinergic agents in combination with analgesics
A03DB − belladonna and derivatives in combination with psycholeptics
A04AD − other antiemetic agents
G04BD − drugs for urinary frequency and incontinence
N04AA − tertiary amines
N04AB − ethers chemically close to antihistamines
N04AC − ethers of tropine or tropine derivatives
S01FA – anticholinergics
R03BB − anticholinergics
R03AL − anticholinergics in combination with adrenergics
**ATC code for drugs with known anticholinergic activity**
A03FA – propulsive agents
A03AX – other drugs for gastrointestinal disorders
C01BA – class IA antiarrhythmics
M03BA − carbamic acid esters
M03BB − oxazole, thiazine and triazine derivatives
M03BC − ethers, chemically close to antihistamines
M03BX − other centrally acting agents
N06AA – non-selective monoamine reuptake inhibitor antidepressants
N05AA − phenothiazines with aliphatic side chain
N05AB – phenothiazines with piperazine structure
N05AC – phenothiazines with piperidine structure
N05AD − butyrophenone derivatives
N05AE − indole derivatives
N05AF – thioxanthen derivatives
N05AG − diphenylbutylpiperidine
N05AH − diazepines, oxazepines, thiazepines and oxepines
N05AL − benzamides
N05AX – other antipsychotics
N05BB – diphenylmethane derivatives
R01BA – sympathomimetics
R06A − antihistamines for systemic use comprising all chemical subgroups (R06AA, R06AB, R06AC, R06AD, R06AE, R06AD, R06AK, R06AX)
2. Inclusion in the previous list of drugs with strong anticholinergic activity referenced in the 2015 American Geriatrics Society Beers Criteria *.* ^(^ ^26^ ^)^
3. Addition of related drugs in at least two anticholinergic scales, with activity grading, identified in the PubMed/MEDLINE^®^ search.
4. Exclusion of drugs not marketed in Brazil, after searching the products duly registered in the country, on the website of the *Agência Nacional de Vigilância Sanitária* (Anvisa).^(^ ^27^ ^)^
5. Exclusion of ophthalmic administration drugs, for diagnostic purposes.
6. Identification of the magnitude of the reported anticholinergic activity for each drug in the scales available. When absent, we searched the drug’s anticholinergic effect profile in Martindale: the complete drug reference^(^ ^18^ ^)^ and assigned a score in comparison with the other drugs in the same class. The scores identified and assigned to the drugs were transformed to be used in this scale, using the system described in previous studies.^(^ ^3^ ^,^ ^5^ ^,^ ^13^ ^)^ 6.1 Evidence of potential serum anticholinergic activity or expert-reported anticholinergic effects. 6.2 Drug with expert-reported, dose-dependent anticholinergic activity. 6.3 Anticholinergic drug or drug with known, expert-reported, strong anticholinergic activity.

Drugs classified as 4 in the scale developed by Ehrt et al.,^(^ ^14^ ^)^ were reclassified as 3. The drugs described by Chew et al.,^(^ ^12^ ^)^ with 0:0 and 0/+ activity were not included in the scale, and we used the following equivalence for the drugs listed by these authors: 1= +, 2= ++ and 3= +++, to describe their anticholinergic activity.

The drugs included in the scale were rated according to the ATC classification, 5^th^ level, chemical substances.

## RESULTS

In the process of developing the scale, we identified 273 drugs with anticholinergic activity, of which 152 are marketed in Brazil. We excluded 25 drugs which were present in only one of the previously selected scales, and two drugs (cyclopentolate and tropicamide) intended for ophthalmic administration. A total of 125 drugs were included in the scale. Of these, 57 were identified in step 1, of which 13 were anticholinergics and 44 had known anticholinergic activity. In step 2, we included a new drug, and 35 of the drugs found at this stage had already been included in the scale. In step 3, we included 67 drugs, of the 115 identified at this stage.

Table 2 displays the Brazilian scale of anticholinergic activity with the respective scores. Of all these drugs, 45 (36.0%) were assigned a score of 3, 13 (10.4%) a score of 2, and 67 (53.6%) a score of 1. Considering the ATC classification, 1^st^ level, anatomical group, we identified that 52 (41.6%) of drugs were for the nervous system, 24 (19.2%) for the respiratory system, 11 (8.8%) for the cardiovascular system, 11 (8.8%) for the digestive tract and metabolism, and 11 (8.8%) were for the genitourinary system and sex hormones. The analysis as per the ATC classification, 3^rd^ level, pharmacological subgroup, showed that antidepressants and antipsychotics accounted for the largest proportion of drugs for the nervous system. Antihistamines for systemic use accounted for the largest proportion of drugs for the respiratory system. For the other systems, there was no predominant pharmacological subgroup.

**Table 2:** Brazilian scale of drugs with anticholinergic activity

Score 3		Score 2		Score 1	
		
ATC	Drug	ATC	Drug	ATC	Drug
A03BA04	Belladonna total alkaloids^*, †,a, c, e, f^	N04BB01	Amantadine^g, i, l, n^	N03AG01	Valproic acid^d, n^
N06AA09	Amitriptyline^b, c, d, e, f, g, h, i, j, k, l, n^	M03BX01	Baclofen^b, f, g, n^	N05BA12	Alprazolam^d, f, i, k, n^
A03BA01	Atropine^a, c, d, f, g, h, i, k, l, n^	N03AF01	Carbamazepine^d, i, n^	J01CA01	Ampicillin^d, n^
N04AA02	Biperiden^a, n^	A02BA01	Cimetidine^d, g, n^	C07AB03	Atenolol^f, i, n^
R06AB01	Brompheniramine^*, b, c, d, i, n^	M03BB03	Chlorzoxazone^*, b^	L04AX01	Azathioprine^d, n^
R06AE01	Buclizine^b^	N05AA02	Cetirizine^f, g, k, n^	G02CB01	Bromocriptine^d, l, n^
A03BB01	Butylscopolamine, bromide^a^	N07BC02	Levomepromazine^b, l, m, n^	N06AX12	Bupropion^f, i, n^
R06AA08	Carbinoxamine^b, c, d, i^	N03AF02	Methadone^f, n^	C09AA01	Captopril^d, i, n^
M03BA02	Carisoprodol^*, b, g^	N02AB02	Oxcarbazepine^d, i, n^	N05AN01	Lithium carbonate^h, k, l, n^
M03BX08	Cyclobenzaprine^b, c, g, h^	N05AG02	Pethidine^d, i, l, n^	J01DC01	Cefoxitin^d, n^
R06AX02	Cyproheptadine^b, c, g, k, l, n^	R01BA02	Pimozide^d, i, l, n^	L04AD01	Cyclosporine^d, n^
R06AA04	Clemastine^b, c, d, i,^	R06AE07	Pseudoephedrine*^,b, g, k, n^	N06AB04	Citalopram^h, k, l, m, n^
N06AA04	Clomipramine^b, c, d, e, i, l, n^	N05AH04	Quetiapine^b, f, j, l, n^	N03AE01	Clonazepam^d, k, m, n^
R06AB04	Chlorpheniramine^b, c, d, e, f, g, i, k, n^			N05BA02	Chordiazepoxide^*, d, f, n^
N05AA01	Chlorpromazine^b,c, d, e, f, g, i, l, n^			C03BA04	Chlorthalidone^d, i, n^
N05AH02	Clozapine^b, c, d, h, i, j, l, n^			R05DA04	Codeine^d, f, i, k, m, n^
G04BD10	Darifenacin^a, c, d, i^			M04AC01	Colchicine^i, n^
N06AA01	Desipramine^b, c d, i^			R06AX27	Desloratadine^b^
R06AB06	Dexbrompheniramine^*, b, c^			H02AB02	Dexamethasone^d, n^
R06AB02	Dexchlorpheniramine^b, c, e, k, n^			N05BA01	Diazepam^d, f, i, j, k, l, m, n^
R06AA02	Diphenhydramine^b, c, d, f, g, i, n^			C01AA05	Digoxin^d, i, j, k, n^
R06AA02	Dimenhydrinate^b, c, d, i, n^			C08DB01	Diltiazem^d, n^
R06AA09	Doxylamine^*, b, c^			B01AC07	Dipyridamole^d, i^
N05AB02	Fluphenazine^b, g, k, n^			A03FA03	Domperidone^b, k, l, m, n^
N05BB01	Hydroxyzine^b, c, d, e, g, i, n^			N04BX02	Entacapone^g, n^
A03BA03	Hyoscyamine^*, a, c, d, g, h, i^			N06AB10	Escitalopram^h, k, m^
A03CB04	Homatropine^a, c, f^			A02BA03	Famotidine^d, n^
N06AA02	Imipramine^b, c, d, e, f, g, i, k, l, n^			N03AA02	Phenobarbital^f, j^
R03BB01	Ipratropium^a, j, n^			N01AH01	Fentanyl^d, i, l, m, n^
N06AA21	Maprotiline^b, e, n^			R06AX26	Fexofenadine^b^
R06AE05	Meclizine^b, c, d, g, i^			N06AB03	Fluoxetine^d, f, h, k, l, m, n^
N06AA10	Nortriptyline^b, c, d, f, i, j, l, n^			N06AB08	Fluvoxamine^d, i, k, n^
N05AH03	Olanzapine^b, c, i, j^			C03CA01	Furosemide^d, i, j^
M03BC01	Orphenadrine*^,b, c, d, e, i, j, l^			J01GB03	Gentamicin^d, n^
G04BD04	Oxybutynin^a, c, d, e, g, i, j, l, n^			N05AD01	Haloperidol^b, g, i, m, n^
N06AB05	Paroxetine^c, i, j^			C02DB02	Hydralazine^d, i^
R06AD02	Promethazine^b, c, d, g, i, l, n^			H02AB09	Hydrocortisone^d, i, n^
G04BD08	Solifenacin^a, c, n^			C01DA08	Isosorbide^d, i, n^
N05AC02	Thioridazine^b, c, d, f, g, h, i, j^			R06AE09	Levocetirizine^b, m^
R03BB04	Tiotropium^a^			N04BA02	Levodopa + carbidopa^f, g, k, n^
M03BX02	Tizanidine^b, g, n^			A07DA03	Loperamide^d, f, i, k, m^
G04BD07	Tolterodine^a, c, d, f, h, i, k, n^			R06AX13	Loratadine^b, f, k^
N04AA01	Trihexyphenidyl^a, c, d, e, f, i, j, l, n^			N05BA06	Lorazepam^d, n^
N05AB06	Trifluoperazine^b, c, g, i^			H02AB04	Methylprednisolone^d, n^
R06AX07	Triprolidine^b, c^			A03FA01	Metoclopramide^b, g, k, n^
				C07AB02	Metoprolol^f, i, n^
				N05CD08	Midazolam^d, n^
				N06AX11	Mirtazapine^g, h, l, m, n^
				N02AA01	Morphine^d, f, i, m, n^
				C08CA05	Nifedipine^d, i, n^
				N02AA05	Oxycodone^d, f, k, m, n^
				J01CA12	Piperacillin^*, d, n^
				N04BC05	Pramipexole^g, n^
				H02AB06	Prednisolone^d, l, n^
				H02AB07	Prednisone^d, i, n^
				A02BA02	Ranitidine^g, h, i, k, m, n^
				N05AX08	Risperidone^b, f, g, i, k, m, n^
				N04BD01	Selegiline^g, n^
				N06AB06	Sertraline^d, f, l, n^
				R03DA04	Theophylline^d, i, m, n^
				N02AX02	Tramadol^d, m, n^
				N02AX05	Trazodone^f, g, i, m, n^
				H02AB08	Triamcinolone^d, n^
				C03DB02	Triamterene^*, d, i, n^
				J01XA01	Vancomycin^d, n^
				B01AA03	Warfarine^d, n^
				N06AX16	Venlafaxine^f, k^

* Drugs marketed only in a combined, fixed dose; ^†^ Fluid extract of Atropa belladona Linné. ^a^Anticholinergic drugs described by Puustinen et al.,^(^ ^24^ ^)^ and Brown et al.;^(^ ^25^ ^)b^drugs with known anticholinergic activity described by Nishtala et al.,^(^ ^2^ ^)^ Salahudeen et al.,^(^ ^8^ ^)^ Hilmer et al.,^(^ ^23^ ^)^ and Puustinen et al.;^(^ ^24^ ^)c^drugs with strong anticholinergic activity described in the 2015 American Geriatrics Society Beers Criteria;^(^ ^26^ ^)d^Anticholinergic Drug Scale;^(^ ^5^ ^)e^Anticholinergic Burden Classification;^(^ ^9^ ^)f^Clinician-Rated Anticholinergic Score;^(^ ^10^ ^)g^Anticholinergic Risk Scale;^(^ ^11^ ^)h^Serum Anticholinergic Activity;^(^ ^12^ ^) i^Anticholinergic Cognitive Burden Scale;^(^ ^13^ ^)j^Anticholinergic Activity Scale;^(^ ^14^ ^)k^Anticholinergic Load Scale;^(^ ^15^ ^)l^Anticholinergic Effect on Cognition;^(^ ^16^ ^)m^Muscarinic Acetylcholinergic Receptor Antagonist Exposure;^(^ ^4^ ^)n^Anticholinergic Impregnation Scale.^(^ ^3^ ^)^

The drugs buclizine, butylscopolamine bromide, dexbrompheniramine, doxylamine, tiotropium, tripolidine, chlorzoxazone and fexofenadine are not present in previously published scales.

## DISCUSSION

The Brazilian scale of anticholinergic activity comprises the drugs available in the country and not listed in other scales, accounting for the specificities of the national market. The number of drugs is close to that of more comprehensive scales, such as the ADS,^(^ ^5^ ^)^ ALS^(^ ^15^ ^)^ and AIS.^(^ ^3^ ^)^

The system applied to develop the scale is simple and allowed us to identify more drugs than a mere compilation of previous scales. Also, the scale can be easily updated through a search of the selected 4^th^ level ATC codes, allowing for an adaptation to the reality of the drugs registered in other countries, or inclusion of new drugs launched in Brazil. More than one third of the scale corresponds to drugs with high anticholinergic activity as per the 2015 American Geriatrics Society Beers Criteria,^(^ ^26^ ^)^ and therefore can be updated through a search of the latest version of said criteria.

In the scale developed, most drugs had an anticholinergic activity score of 1. However, drugs with low anticholinergic activity must be considered, since the toxicity of anticholinergics very often results from a cumulative anticholinergic burden rather than the effect of one single drug.^(^ ^28^ ^)^ An Australian study of community-living elderly, with and without dementia, identified, in both groups, that level 1 drugs were the greatest contributors to the anticholinergic burden (64 to 70%), followed by level 3 drugs (20 to 29%) and level 2 drugs, which contributed with less than 10% of total anticholinergic burden of the population.^(^ ^29^ ^)^

The knowledge healthcare professionals have about potentially hazardous drugs is very limited, as well as their knowledge about drugs with anticholinergic activity. A British study showed that only 37% of healthcare professionals investigated were able to evaluate the anticholinergic burden.^(^ ^29^ ^)^ In view of this, having national scales to rate the anticholinergic activity of different drugs is important to optimize prescription and improve the safety of drug therapy.

Multidisciplinary and/or interdisciplinary work can contribute to decrease the number of drugs with anticholinergic activity. In a study with psychiatric patients, interventions jointly performed by physicians and pharmacists helped decrease the anticholinergic burden, leading to significantly improved memory and quality of life for patients.^(^ ^30^ ^)^

Measurement of the serum anticholinergic activity is expensive and not available in most healthcare services. Therefore, the development of a national anticholinergic activity scale is an useful and practical strategy for healthcare professionals, which may contribute to clinical decision-making, guiding the selection and prescription of safer drugs, helping identify patients with a greater risk of adverse reactions due to anticholinergic burden. Moreover, it contributes to pharmacoepidemiology research, providing more accurate measurements and enhancing knowledge about the impacts of anticholinergic overload on health outcomes of older adults, psychiatric patients and those with Parkinson disease.^(^ ^4^ ^,^ ^5^ ^,^ ^11^ ^)^

The measurement of anticholinergic activity using scales shows great variability due to the lack of consensus regarding the drugs included and the ranking of scores. The system used to develop the Brazilian scale, based on the ATC classification and therapeutic groups related with anticholinergic drugs, as well as drugs with known anticholinergic activity and high anticholinergic activity as described in the 2015 American Geriatrics Society Beers Criteria,^(^ ^26^ ^)^ is in line with the recommendation to standardize scales and adapt them to national markets.^(^ ^1^ ^)^

The Brazilian scale has limitations, since it does not include dose-related information, a recommendation already incorporated into the MARANTE scale.^(^ ^4^ ^)^ Adding dose-related information to scales allows for a more accurate assessment of the exposure to anticholinergic drugs. The dose is important, particularly in the elderly, due to pharmacokinetic and pharmacodynamic changes related to aging. The administration route was not included either, however it is important to clarify that ophthalmic administration drugs were excluded when used for diagnostic purposes, since this does not characterize continued exposure. Another limitation is that the bibliographic search was restricted to Pubmed/MEDLINE^®^ and the English language. However, our comprehensive search strategy allowed for identification of a large number of scales and three systematic reviews.

The validation of this scale in different healthcare settings is the next step, as well as comparison with the scales currently available. Other investigation perspectives must contemplate the inclusion of information on the minimum effective dose of the drugs in this scale, as well as blood-brain barrier permeability, P-glycoprotein transmembrane transport regulation, and the subtypes of receptor they act upon. It is also essential to investigate ways to standardize the scores of the drugs included in anticholinergic activity lists.

## CONCLUSION

The methodology used to develop the Brazilian anticholinergic activity scale is simple and systematized. The scale of 125 drugs accounts for the specificities of the Brazilian pharmaceutical market and enables assessing the impact of anticholinergic burden of drugs on health outcomes, particularly in older adults, psychiatric patients and those with Parkinson disease. Moreover, this scale may contribute to pharmacoepidemiology research leading to more accurate measurements of anticholinergic activity.
